# Formalized Proofs of the Infinity and Normal Form Predicates in the First-Order Theory of Rewriting

**DOI:** 10.1007/978-3-030-45237-7_11

**Published:** 2020-03-13

**Authors:** Alexander Lochmann, Aart Middeldorp

**Affiliations:** 8 Johannes Kepler University, Linz, Austria; 9 University of Birmingham, Birmingham, UK; Department of Computer Science, University of Innsbruck, Innsbruck, Austria

**Keywords:** Formalization, First-order theory of rewriting, Tree automata

## Abstract

We present a formalized proof of the regularity of the infinity predicate on ground terms. This predicate plays an important role in the first-order theory of rewriting because it allows to express the termination property. The paper also contains a formalized proof of a direct tree automaton construction of the normal form predicate, due to Comon.

## 1 Introduction

Term rewriting [1,18] is an abstract model of computation which underlies much of declarative programming and automated theorem proving. The foundation of rewriting is equational logic. Equations are used from left to right to direct the search for proofs. Fundamental properties like *confluence* (which ensures that different computation paths produce the same result) and *termination* (all computation paths produce a result) are *undecidable* in general. For terminating systems, one is interested in estimating the resources needed to evaluate expressions (space and time *complexity*). Much progress has been made in establishing sufficient and automatable criteria for confluence, termination, complexity, and other properties of rewrite systems. These criteria have been implemented in highly optimized automatic tools that compete on a yearly basis [12,13]. These competitions, together with the recent advances in SAT [4] and SMT [2] solving, have on the one hand led to specialized techniques that are especially suitable for automation. On the other hand, software bugs observed in the tools gave rise to the more recent activity of *certification* of the output of termination, complexity, and confluence tools. This is done by formalizing the underlying methods in an interactive proof assistant like Coq [3] or Isabelle [15], and using the code generation facilities of these proof assistants to obtain trustworthy programs that can certify the output of the tools.

In this paper we are concerned with the formalization of methods that are used in FORT [16,17], a tool that implements the first-order theory of rewriting

* This research is supported by FWF (Austrian Science Fund) project P30301.

for the decidable class of left-linear, right-ground rewrite systems. FORT can be used to decide properties of a given rewrite system and to synthesize rewrite systems that satisfy arbitrary properties expressible in the first-order theory of rewriting. The decision procedure is based on tree automata techniques and goes back to a paper by Dauchet and Tison [7]. In a recent paper [10] the authors formalized results concerning ground tree transducers and $\mathrm{RR}_n$ automata for a fragment of the first-order theory that allows to express confluence, resulting in a formalized confluence prover for left-linear, right-ground rewrite systems. In this paper we cover the infinity predicate that is crucial for expressing the termination property in the first-order theory of rewriting and an efficient automaton construction of the normal form predicate that is employed in FORT. The former goes back to a technical report by Dauchet and Tison [8] and the latter is based on a paper by Comon [5]. The normal form predicate has other applications as well (e.g. [9,14]). A proof of the construction of [8] is given in [16], but this proof contains a serious mistake that we report at the end of Section 3.

Our formalizations are based on IsaFoR [19],[1] an Isabelle/HOL library containing numerous abstract results and concrete techniques from the rewriting literature. Our own development can be found at http://cl-informatik.uibk.ac.at/software/fortissimo/tacas2020/

Most definitions, theorems, and lemmata in this paper directly correspond to the formalization. These are indicated by the ☑ symbol, which links to a HTML presentation in the PDF version of the paper.

In the next section we recall basic definitions, notation, and results concerning term rewriting and tree automata that we need in the sequel. In Section 3 we present our first main result, a formalized correctness proof of the regularity of the infinity predicate for regular relations. The tree automaton constructed in the correctness proof is not directly executable due to the definition of $Q_\infty$ which plays an important role in the construction of the tree automaton. In Section 4 we present our second main result, an equivalent definition of $Q_\infty$ that is constructive. Our third result, a formalized correctness proof of an efficient tree automata construction of the normal form predicate for left-linear rewrite systems, is the topic of Section 5. We conclude in Section 6 with some statistics of our formalizations as well as a list of tasks that remain to be done for a certified version of FORT.

When we write "formalized" we always mean "formalized in Isabelle/HOL."

## 2 Preliminaries

Familiarity with term rewriting [1] and tree automata [6] is useful, but we briefly recall important definitions and notation that we use in the remainder.

We assume a given signature $\mathcal{F}$ and a set of variables $\mathcal{V}$. Function symbols in $\mathcal{F}$ are equipped with a fixed arity. Function symbols of arity zero are called

[1] http://cl-informatik.uibk.ac.at/isafor/

constants. The set of terms built from $\mathcal{F}$ and $\mathcal{V}$ is denoted by $\mathcal{T}(\mathcal{F},\mathcal{V})$ and inductively defined: A term is either a variable $x \in \mathcal{V}$ or $f(t_1,\ldots,t_n)$ for a function symbol $f$ of arity $n$ and terms $t_1,\ldots,t_n \in \mathcal{T}(\mathcal{F},\mathcal{V})$. The set of variables occurring in a term $t$ is denoted by $\mathcal{V}\mathsf{ar}(t)$. A term $t$ with $\mathcal{V}\mathsf{ar}(t) = \varnothing$ is called ground. We write $\mathcal{T}(\mathcal{F})$ for the set of ground terms. Positions are strings of positive integers which are used to address subterms. The empty string is called root position and denoted by $\epsilon$. The set of positions in a term $t$ is denoted by $\mathcal{P}\mathsf{os}(t)$ and the subterm of $t$ at position $p \in \mathcal{P}\mathsf{os}(t)$ by $t|_p$. We write $s \lhd t$ if $s$ is a proper subterm of $t$, i.e., $s = t|_p$ with $p \neq \epsilon$. We write $t[u]_p$ for the result of replacing the subterm of $t$ at position $p$ with the term $u$. The root symbol of a term $t$ is denoted by $\mathsf{root}(t)$ and $t(p)$ denotes $\mathsf{root}(t|_p)$. We write $p < q$ if $p$ is a proper prefix of $q$. A context $C$ is a term with a hole $\Box$. Here $\Box \notin \mathcal{F}$ is a special constant. We write $C[t]$ for the result of replacing the hole in $C$ by $t$. A substitution $\sigma$ is a mapping from variables to terms. We write $t\sigma$ for the result of applying $\sigma$ to the term $t$.

A term rewrite system (TRS for short) $\mathcal{R}$ consists of rewrite rules $\ell \to r$ between terms $\ell$ and $r$ over the same signature $\mathcal{F}$ such that $\mathcal{V}\mathsf{ar}(r) \subseteq \mathcal{V}\mathsf{ar}(\ell)$. The rewrite relation $\to_{\mathcal{R}}$ is defined on terms as follows: $s \to_{\mathcal{R}} t$ if there exist a position $p \in \mathcal{P}\mathsf{os}(s)$, a rewrite rule $\ell \to r \in \mathcal{R}$, and a substitution $\sigma$ such that $s|_p = \ell\sigma$ and $t = s[r\sigma]_p$. The reflexive transitive closure of $\to_{\mathcal{R}}$ is denoted by $\to_{\mathcal{R}}^*$. A redex is a substitution instance of a left-hand side of a rewrite rule. Terms that contain a redex as subterm are called reducible. A normal form is a term without redexes. We write $\mathsf{NF}(\mathcal{R})$ for the set of ground normal forms of $\mathcal{R}$. In this paper we consider finite TRSs over finite signatures. The TRSs handled by FORT are left-linear (no duplicate variables in left-hand sides of rewrite rules) and right-ground (no variables in right-hand sides of rewrite rules).

We now recall some basic notions related to tree automata. A *tree automaton* is a quadruple $\mathcal{A} = (\mathcal{F}, Q, Q_f, \Delta)$ consisting of a finite signature $\mathcal{F}$, a finite set $Q$ of states, disjoint from $\mathcal{F}$, a subset $Q_f \subseteq Q$ of final states, and a set of transition rules $\Delta$. Every transition rule has one of the following two shapes:

- $f(p_1,\ldots,p_n) \to q$ with $f \in \mathcal{F}$ and $p_1,\ldots,p_n,q \in Q$, or
- $p \to q$ with $p,q \in Q$.

Transition rules of the second shape are called epsilon transitions. We write $\Delta_\epsilon$ for the set of epsilon transitions. Furthermore, $\Delta_{\neg\epsilon} = \Delta \setminus \Delta_\epsilon$. Transition rules can be viewed as rewrite rules between ground terms in $\mathcal{T}(\mathcal{F} \cup Q)$. The induced rewrite relation is denoted by $\to_\Delta$ or $\to_{\mathcal{A}}$. A ground term $t \in \mathcal{T}(\mathcal{F})$ is *accepted* by $\mathcal{A}$ if $t \to_\Delta^* q$ for some $q \in Q_f$. The set of all accepted terms is denoted by $L(\mathcal{A})$ and a set $L$ of ground terms is *regular* if $L = L(\mathcal{A})$ for some tree automaton $\mathcal{A}$.

Let $\mathcal{A} = (\mathcal{F}, Q, Q_f, \Delta)$ be a tree automaton. A state $q \in Q$ is *reachable* if $t \to_\Delta^* q$ for some term $t \in \mathcal{T}(\mathcal{F})$. We say that $q$ is *productive* if $C[q] \to_\Delta^* q_f$ for some ground context $C$ and final state $q_f \in Q_f$. The automaton $\mathcal{A}$ is *trim* if all states are both reachable and productive. Any tree automaton can be transformed into an equivalent trim automaton. This result has been formalized in IsaFoR by Felgenhauer and Thiemann [11].

Below we present a formalized proof of a version of the *pumping lemma* that we need later.

**Lemma 1.** *Let* $\mathcal{A} = (\mathcal{F}, Q, Q_f, \Delta)$ *be a tree automaton and* $t \to_\Delta^* q$ *with* $t \in \mathcal{T}(\mathcal{F})$ *and* $q \in Q$*. If* $\mathsf{height}(t) > |Q|$ *then there exist contexts* $C_1$ *and* $C_2 \neq \square$*, a term* $u$*, and a state* $p$ *such that* $t = C_1[C_2[u]]$*,* $u \to_\Delta^* p$*,* $C_2[p] \to_\Delta^* p$*, and* $C_1[p] \to_\Delta^* q$*.*

*Proof.* From the assumptions $t \to_\Delta^* q$ and $\mathsf{height}(t) > |Q|$ we obtain a sequence $(t_1, \ldots, t_{n+1}, q_1, \ldots, q_{n+1}, D_1, \ldots, D_n)$ consisting of ground terms, states, and non-empty contexts with $n > |Q|$ such that

- $t_i \to_\Delta^* q_i$ for all $i \leqslant n+1$,
- $D_i[t_i] = t_{i+1}$ and $D_i[q_i] \to_\Delta^* q_{i+1}$ for all $i \leqslant n$, and
- $q_{n+1} = q$ and $t_{n+1} = t$

by a straightforward induction proof on $t$. Because $n > |Q|$ there exist indices $1 \leqslant i < j \leqslant n$ such that $q_i = q_j$. We construct the contexts $C_1 = D_n[\ldots[D_j]\ldots]$ and $C_2 = D_{j-1}[\ldots[D_i]\ldots]$. Note that $C_2 \neq \square$ as $i < j$. We obtain $C_2[q_i] \to_\Delta^* q_j$ and $C_1[q_j] \to_\Delta^* q_{n+1}$ by induction on the difference $j - i$. By letting $p = q_i = q_j$ and $u = t_i$ we obtain the desired result. ☑

We conclude this preliminary section with a brief account of $\mathrm{RR}_2$ relations, which are binary relations on ground terms over a signature $\mathcal{F}$ whose *encoding* as sets of ground terms over the extended signature $\mathcal{F}^{(2)} = (\mathcal{F} \cup \{\bot\})^2$ with a fresh constant $\bot \notin \mathcal{F}$ is regular. The arity of a symbol $fg \in \mathcal{F}^{(2)}$ is the maximum of the arities of $f$ and $g$. The encoding of two terms $t, u \in \mathcal{T}(\mathcal{F})$ is the unique term $\langle t, u\rangle \in \mathcal{T}(\mathcal{F}^{(2)})$ such that $\mathcal{P}\mathsf{os}(\langle t, u\rangle) = \mathcal{P}\mathsf{os}(t) \cup \mathcal{P}\mathsf{os}(u)$ and $\langle t, u\rangle(p) = fg$ where

$$f = \begin{cases} t(p) & \text{if } p \in \mathcal{P}\mathsf{os}(t) \\ \bot & \text{otherwise} \end{cases} \qquad\qquad g = \begin{cases} u(p) & \text{if } p \in \mathcal{P}\mathsf{os}(u) \\ \bot & \text{otherwise} \end{cases}$$

for all positions $p \in \mathcal{P}\mathsf{os}(t) \cup \mathcal{P}\mathsf{os}(u)$. We illustrate this on a concrete example. For the ground terms $t = \mathsf{f}(\mathsf{g}(\mathsf{a}), \mathsf{f}(\mathsf{b}, \mathsf{a}))$ and $u = \mathsf{f}(\mathsf{a}, \mathsf{g}(\mathsf{g}(\mathsf{b})))$ we obtain $\langle t, u\rangle = \mathsf{ff}(\mathsf{ga}(\mathsf{a}\bot), \mathsf{fg}(\mathsf{bg}(\bot\mathsf{b}), \mathsf{a}\bot))$. A tree automaton operating on terms in $\mathcal{T}(\mathcal{F}^{(2)})$ is called an $\mathrm{RR}_2$ automaton. The two *projection* operations effectively transform $\mathrm{RR}_2$ relations on $\mathcal{T}(\mathcal{F})$ to regular subsets of $\mathcal{T}(\mathcal{F})$.

## 3 Infinity Predicate

The following formula in the first-order theory of rewriting expresses the termination property:[2]

$$\forall\, t\ \mathsf{FIN}_{\to^+}(t) \,\wedge\, \neg\, \exists\, u\, (u \to^+ u)$$

[2] The formula characterizes termination of all rewrite systems $\mathcal{R}$ with the property that the induced rewrite relation $\to_\mathcal{R}$ is *finitely branching*.

The predicate $\mathsf{FIN}_{\to^+}$ holds for $t \in \mathcal{T}(\mathcal{F})$ if there are only finitely many terms $u \in \mathcal{T}(\mathcal{F})$ such that $t \to^+ u$. We consider its complement as it leads to smaller automata:

$$\neg\,\exists\, t\ (\mathsf{INF}_{\to^+}(t) \vee t \to^+ t)$$

with $\mathsf{INF}_{\to^+} = \{t \in \mathcal{T}(\mathcal{F}) \mid t \to_{\mathcal{R}}^+ u \text{ for infinitely many terms } u \in \mathcal{T}(\mathcal{F})\}$.

**Definition 1.** *Let* $\circ$ *be an arbitrary binary relation on* $\mathcal{T}(\mathcal{F})$*. We write* $\mathsf{INF}_\circ$ *for the set* $\{t \in \mathcal{T}(\mathcal{F}) \mid (t,u) \in \circ$ *for infinitely many terms* $u \in \mathcal{T}(\mathcal{F})\}$*.*

In [8] the construction of a tree automaton that accepts $\mathsf{FIN}_\circ$ for an arbitrary $\mathrm{RR}_2$ relation $\circ$[3] is given. In [16, Appendix A] a correctness proof of the construction is presented, which contains a serious mistake (reported at the end of this section). In this section we give a rigorous and formalized proof of the regularity of $\mathsf{INF}_\circ$ for arbitrary $\mathrm{RR}_2$ relations $\circ$.

**Theorem 1.** *The set* $\mathsf{INF}_\circ$ *is regular for every* $RR_2$ *relation* $\circ \subseteq \mathcal{T}(\mathcal{F}) \times \mathcal{T}(\mathcal{F})$*.*

The following definition originates from [8].

**Definition 2.** *Given a tree automaton* $\mathcal{A} = (\mathcal{F}^{(2)}, Q, Q_f, \Delta)$*, the set* $Q_\infty \subseteq Q$ *consists of all states* $q \in Q$ *such that* $\langle \bot, t\rangle \to_\Delta^* q$ *for infinitely many terms* $t \in \mathcal{T}(\mathcal{F})$*.*

*Example 1.* Consider the binary relation

$$\circ = \{(\mathsf{f}(\mathsf{a},\mathsf{g}^n(\mathsf{b})), \mathsf{g}^m(\mathsf{f}(\mathsf{a},\mathsf{b}))) \mid n = 2 \text{ and } m \geqslant 1 \text{ or } n \geqslant 3 \text{ and } m = 1\}$$

The encoding of $\circ$ is accepted by the $\mathrm{RR}_2$ automaton $\mathcal{A} = (\mathcal{F}^{(2)}, Q, Q_f, \Delta)$ with $\mathcal{F} = \{\mathsf{a},\mathsf{b},\mathsf{f},\mathsf{g}\}$, $Q = \{0,\dots,11\}$, $Q_f = \{0\}$, and $\Delta$ consisting of the following transition rules:

$$\begin{array}{llll}
\mathsf{fg}(1,2) \to 0 & \bot\mathsf{f}(3,4) \to 5 & \mathsf{g}\bot(6) \to 2 & \mathsf{b}\bot \to 7 \\
\mathsf{fg}(8,9) \to 0 & \bot\mathsf{g}(5) \to 5 & \mathsf{g}\bot(7) \to 6 & \mathsf{b}\bot \to 11 \\
\mathsf{af}(3,4) \to 1 & \bot\mathsf{a} \to 3 & \mathsf{g}\bot(10) \to 9 & \mathsf{ag}(5) \to 1 \\
\mathsf{af}(3,4) \to 8 & \bot\mathsf{b} \to 4 & \mathsf{g}\bot(11) \to 10 & \mathsf{g}\bot(11) \to 11
\end{array}$$

For instance,

$$\begin{aligned}
\langle \mathsf{f}(\mathsf{a},\mathsf{g}(\mathsf{g}(\mathsf{b}))), \mathsf{g}(\mathsf{f}(\mathsf{a},\mathsf{b}))\rangle &= \mathsf{fg}(\mathsf{af}(\bot\mathsf{a},\bot\mathsf{b}), \mathsf{g}\bot(\mathsf{g}\bot(\mathsf{b}\bot))) \\
&\to_\Delta^* \mathsf{fg}(\mathsf{af}(3,4), \mathsf{g}\bot(\mathsf{g}\bot(7))) \to_\Delta^* \mathsf{fg}(1, \mathsf{g}\bot(6)) \to_\Delta \mathsf{fg}(1,2) \to_\Delta 0
\end{aligned}$$

but $\langle \mathsf{f}(\mathsf{a},\mathsf{g}(\mathsf{b}), \mathsf{f}(\mathsf{a},\mathsf{b}))\rangle = \mathsf{ff}(\mathsf{aa}, \mathsf{gb}(\mathsf{b}\bot))$ is not accepted.

We have $Q_\infty = \{5\}$. State 5 is reached by $\langle \bot, \mathsf{g}^n(\mathsf{f}(\mathsf{a},\mathsf{b}))\rangle$ for all $n \geqslant 0$.

[3] The relation $\to_{\mathcal{R}}^+$ is an $\mathrm{RR}_2$ relation for left-linear, right-ground TRSs $\mathcal{R}$. Other uses of $\mathsf{FIN}$ ($\mathsf{INF}$) can be found in [16].

**Definition 3.** *Given a tree automaton $\mathcal{A} = (\mathcal{F}^{(2)}, Q, Q_f, \Delta)$, we define the tree automaton $\mathcal{A}_\infty = (\mathcal{F}^{(2)}, Q \cup \bar{Q}, \bar{Q}_f, \Delta \cup \bar{\Delta})$. Here $\bar{Q}$ is a copy of $Q$ where every state is dashed: $\bar{q} \in \bar{Q}$ if and only if $q \in Q$. For every transition rule $fg(q_1, \ldots, q_n) \to q \in \Delta$ we have the following transition rules in $\bar{\Delta}$:*

$$fg(q_1, \ldots, q_n) \to \bar{q} \qquad \textit{if } q \in Q_\infty \textit{ and } f = \bot \tag{1}$$

$$fg(q_1, \ldots, q_{i-1}, \bar{q}_i, q_{i+1}, \ldots, q_n) \to \bar{q} \qquad \textit{for all } 1 \leqslant i \leqslant n \tag{2}$$

*Moreover, for every $\epsilon$-transition $p \to q \in \Delta$ we add*

$$\bar{p} \to \bar{q} \tag{3}$$

*to $\bar{\Delta}$. We write $\Delta'$ for $\Delta \cup \bar{\Delta}$.*

Dashed states are created by rules of shape (1) and propagated by rules of shapes (2) and (3). The above construction differs from the one in [8]; instead of (1) the latter contains $fg(q_1, \ldots, q_n) \to \bar{q}$ if $q_i \in Q_\infty$ for some $i > \mathsf{arity}(f)$. In an implementation, rather than adding all dashed states and all transition rules of shape (2), the necessary rules would be computed by propagating the dashes created by (1) in order to avoid the appearance of unreachable dashed states. When $\mathcal{A}_\infty$ is used in isolation, a single bit suffices to record that a dashed state occurred during a computation.

*Example 2.* For the tree automaton $\mathcal{A}$ from Example 1 we obtain $\mathcal{A}_\infty$ by adding the following transition rules (the missing rules of shape (2) involve unreachable states):

$$\bot\mathsf{f}(3,4) \to \bar{5} \qquad \bot\mathsf{g}(5) \to \bar{5} \qquad \bot\mathsf{g}(\bar{5}) \to \bar{5} \qquad \mathsf{ag}(\bar{5}) \to \bar{1} \qquad \mathsf{fg}(\bar{1},2) \to \bar{0}$$

The unique final state of $\mathcal{A}_\infty$ is $\bar{0}$. We have $\langle \mathsf{f}(\mathsf{a}, \mathsf{g}(\mathsf{g}(\mathsf{b}))), \mathsf{g}(\mathsf{f}(\mathsf{a}, \mathsf{b})) \rangle \in L(\mathcal{A}_\infty)$ but there is no term $u$ such that $\langle \mathsf{f}(\mathsf{a}, \mathsf{g}(\mathsf{b})), u \rangle \in L(\mathcal{A}_\infty)$.

The following preliminary lemma is proved by a straightforward induction argument.

**Lemma 2.** *If $t \to_{\mathcal{A}}^* p$ then $t \to_{\mathcal{A}_\infty}^* p$. If $C[p] \to_{\mathcal{A}}^* q$ then $C[p] \to_{\mathcal{A}_\infty}^* q$ and $C[\bar{p}] \to_{\mathcal{A}_\infty}^* \bar{q}$.*

**Theorem 2.** *Suppose $\circ$ is accepted by the $RR_2$ automaton $\mathcal{A}$. If $t \in \mathsf{INF}_\circ$ then $\langle t, u \rangle \in L(\mathcal{A}_\infty)$ for some term $u \in \mathcal{T}(\mathcal{F})$.*

*Proof.* From $t \in \mathsf{INF}_\circ$ and $\circ = L(\mathcal{A})$ we obtain $\langle t, u \rangle \in L(\mathcal{A})$ for infinitely many terms $u \in \mathcal{T}(\mathcal{F})$. Since the signature is finite, there are only finitely many ground terms of any given height. Moreover, $\mathsf{height}(\langle t, u \rangle) = \max(\mathsf{height}(t), \mathsf{height}(u))$. Hence there must exist a term $u \in \mathcal{T}(\mathcal{F})$ with $\langle t, u \rangle \in L(\mathcal{A})$ such that

$$\mathsf{height}(t) + |Q| + 1 < \mathsf{height}(u)$$

This is only possible if there are positions $p$ and $q$ such that $p \notin \mathcal{P}\mathsf{os}(t)$, $pq \in \mathcal{P}\mathsf{os}(u)$, and $|Q| < |q|$. From $\mathcal{P}\mathsf{os}(\langle t,u\rangle) = \mathcal{P}\mathsf{os}(t) \cup \mathcal{P}\mathsf{os}(u)$ we obtain $\langle t,u\rangle|_p = \langle \bot, u|_p\rangle$. Since $\langle t,u\rangle \in L(\mathcal{A})$ there exist states $r \in Q$ and $q \in Q_f$ such that

$$\langle t,u\rangle = \langle t,u\rangle[\langle \bot, u|_p\rangle]_p \to_{\mathcal{A}}^* \langle t,u\rangle[r]_p \to_{\mathcal{A}}^* q_f$$

where we assume without loss of generality that the last step in the subsequence $\langle \bot, u|_p\rangle \to_{\mathcal{A}}^* r$ uses a non-epsilon transition rule.

From $|Q| < |q|$ and $pq \in \mathcal{P}\mathsf{os}(u)$ we infer $|Q| < \mathsf{height}(\langle \bot, u|_p\rangle)$. Hence we can use the pumping lemma (Lemma 1) to conclude the existence of infinitely many terms $v \in \mathcal{T}(\mathcal{F})$ such that $\langle \bot, v\rangle \to_{\mathcal{A}}^* r$. Hence $r \in Q_\infty$ by Definition 2. Since the last step $\langle \bot, u|_p\rangle \to_{\mathcal{A}}^* r$ uses a non-epsilon transition rule, we obtain $\langle \bot, u|_p\rangle \to_{\mathcal{A}_\infty}^* \bar{r}$ using Lemma 2 and a final application of a rule of shape (1). Also using Lemma 2 we obtain $\langle t,u\rangle[\bar{r}]_p \to_{\mathcal{A}_\infty}^* \bar{q}_f$ as $\langle t,u\rangle[r]_p \to_{\mathcal{A}}^* q_f$. We conclude $\langle t,u\rangle \in L(\mathcal{A}_\infty)$ as desired. ☑

For the reverse direction of Theorem 3 we need two auxiliary results. The first result is proved by a straightforward induction argument. Here the mapping $\varphi\colon \mathcal{T}(\mathcal{F}^{(2)} \cup Q \cup \bar{Q}) \to \mathcal{T}(\mathcal{F}^{(2)} \cup Q)$ erases all dashes from states.

**Lemma 3.** *If $t \in \mathcal{T}(\mathcal{F}^{(2)} \cup Q \cup \bar{Q})$ and $t \to_{\mathcal{A}_\infty}^* p$ then $\varphi(t) \to_{\mathcal{A}}^* \varphi(p)$.* ☑

With a little bit more effort, we obtain the second auxiliary result. The key step in the proof is identifying the rule of shape (1) that is used to create the first dashed state.

**Lemma 4.** *If $t \in \mathcal{T}(\mathcal{F}^{(2)})$ and $t \to_{\mathcal{A}_\infty}^* \bar{p}$ then there exist a state $q \in Q_\infty$, a context $C$, and a term $s$ such that $C[s] = t$, $\mathsf{root}(s) = \bot f$ for some $f \in \mathcal{F}$, $s \to_{\mathcal{A}_\infty}^* \bar{q}$, and $C[\bar{q}] \to_{\mathcal{A}_\infty}^* \bar{p}$.* ☑

**Theorem 3.** *Suppose $\circ$ is accepted by the RR$_2$ automaton $\mathcal{A}$. If $\langle t,u\rangle \in L(\mathcal{A}_\infty)$ for some term $u \in \mathcal{T}(\mathcal{F})$ then $t \in \mathsf{INF}_\circ$.*

*Proof.* From $\langle t,u\rangle \in L(\mathcal{A}_\infty)$ we obtain a final state $\bar{q}_f \in \bar{Q}$ with $\langle t,u\rangle \to_{\mathcal{A}_\infty}^* \bar{q}_f$. Using Lemma 4, we obtain a context $C$, a term $s$ with $\mathsf{root}(s) = \bot f$ for some $f \in \mathcal{F}$, and a state $q \in Q_\infty$ such that $C[s] = \langle t,u\rangle$, $s \to_{\mathcal{A}_\infty}^* \bar{q}$, and $C[\bar{q}] \to_{\mathcal{A}_\infty}^* \bar{q}_f$. Let $p$ be the position of the hole in $C$. From $C[s] = \langle t,u\rangle$ and $\mathsf{root}(s) = \bot f$, we infer $p \in \mathcal{P}\mathsf{os}(u) \setminus \mathcal{P}\mathsf{os}(t)$. Since $q \in Q_\infty$ the set $\{v \in \mathcal{T}(\mathcal{F}) \mid \langle \bot, v\rangle \to_{\mathcal{A}}^* q\}$ is infinite. Hence the set $S = \{u[v]_p \in \mathcal{T}(\mathcal{F}) \mid \langle \bot, v\rangle \to_{\mathcal{A}}^* q\}$ is infinite, too. Let $u[w]_p \in S$. So $\langle \bot, w\rangle \to_{\mathcal{A}}^* q$. Since $C$ is ground and $C[\bar{q}] \to_{\mathcal{A}_\infty}^* \bar{q}_f$, we obtain $C[q] \to_{\mathcal{A}}^* q_f$ from Lemma 3. We have $C[w] = \langle t, u[w]_p\rangle$ as $p \in \mathcal{P}\mathsf{os}(u) \setminus \mathcal{P}\mathsf{os}(t)$. It follows that $\langle t, u[w]_p\rangle \in L(\mathcal{A})$ and thus there are infinitely many terms $u$ such that $\langle t,u\rangle \in L(\mathcal{A})$. Since $\circ = L(\mathcal{A})$ we conclude the desired $t \in \mathsf{INF}_\circ$. ☑

The final step to conclude that the infinity predicate is indeed regular is now easy.

*Proof (of Theorem 1).* Combining Theorem 2 and Theorem 3 yields the following equivalence:

$$t \in \mathsf{INF}_\circ \quad\Longleftrightarrow\quad \langle t, u\rangle \in L(\mathcal{A}_\infty) \text{ for some term } u$$

Hence a tree automaton that accepts $\mathsf{INF}_\circ$ is obtained by subjecting $\mathcal{A}_\infty$ to a projection operation (on the first argument). □

Projection on $\mathrm{RR}_n$ automata has been formalized in Isabelle/HOL as part of [10].

The mistake in the proof given in the appendix of [16] is quoted below and corresponds to the proof of Theorem 2:

> The set $\mathcal{U} = \{u \in \mathcal{T}(\mathcal{F}) \mid (t, u) \in \circ\}$ is infinite. Since the signature $\mathcal{F}$ is finite, infinitely many terms $u$ in $\mathcal{U}$ have a height greater than $t$. Hence there exists a position $p \notin \mathcal{P}\mathsf{os}(t)$ such that the set $\mathcal{U}' = \{u \in \mathcal{U} \mid p \in \mathcal{P}\mathsf{os}(u)\}$ is infinite. For every $u \in \mathcal{U}'$ we have $\langle t, u\rangle|_p = \langle \bot, u|_p\rangle$. Since $\langle t, u\rangle$ is accepted by $\mathcal{A}$ and $Q$ is finite, there must exist a state $q'$ such that $\langle \bot, u|_p\rangle \to^*_{\mathcal{A}} q'$ for infinitely many terms $u \in \mathcal{U}'$. Therefore $q' \in Q_\infty$.

The following example refutes the above reasoning, which is the key step in the proof in [16]. It was found in attempt to formalize the proof.

*Example 3.* Let $t = \mathsf{f}(\mathsf{a}, \mathsf{b})$ and consider the infinite set $\mathcal{U} = \{\mathsf{f}(\mathsf{f}(\mathsf{a}, \mathsf{b}), \mathsf{g}^n(\mathsf{b})) \mid n \geqslant 1\}$. The automaton

$$\mathcal{A} = (\{\mathsf{f}, \mathsf{g}, \mathsf{a}, \mathsf{b}\}^{(2)}, \{q_1, \ldots, q_6\}, q_6, \Delta)$$

with $\Delta$ consisting of the transition rules

$$\begin{array}{llll} \mathsf{ff}(q_4, q_5) \to q_6 & \bot\mathsf{a} \to q_2 & \mathsf{bg}(q_1) \to q_5 & \bot\mathsf{b} \to q_1 \\ \mathsf{af}(q_2, q_3) \to q_4 & \bot\mathsf{b} \to q_3 & \bot\mathsf{g}(q_1) \to q_1 & \end{array}$$

accepts the relation $\circ = \{t\} \times \mathcal{U}$. Consider the position $p = 11$. We have $p \notin \mathcal{P}\mathsf{os}(t)$ and $p \in \mathcal{P}\mathsf{os}(u)$ for all terms $u \in \mathcal{U}$. Hence $\mathcal{U}' = \mathcal{U}$. Moreover, $\langle t, u\rangle|_p = \langle \bot, \mathsf{a}\rangle = \bot\mathsf{a}$ for all terms $u \in \mathcal{U}'$. The only state reachable from $\bot\mathsf{a}$ is $q_2$ and clearly $q_2 \notin Q_\infty$.

## 4 Executable Infinity Predicate

Owing to the definition of $Q_\infty$, the automaton $\mathcal{A}_\infty$ defined in Definition 3 is not executable. In this section we give an equivalent but executable definition of $Q_\infty$, which we name $Q^e_\infty$:

$$Q^e_\infty = \{q \mid p \rightsquigarrow p \text{ and } p \rightsquigarrow q \text{ for some state } p \in Q\}$$

Here the relation $\rightsquigarrow$ is defined using the inference rules in Figure 1. Before proving that the two definitions are equivalent, we illustrate the definition of $Q^e_\infty$ by revisiting Example 1.

$$\frac{\bot f(p_1,\ldots,p_n) \to_\Delta p}{p_1 \rightsquigarrow p \quad \cdots \quad p_n \rightsquigarrow p} \qquad \frac{p \rightsquigarrow q \quad q \to_\Delta r}{p \rightsquigarrow r} \qquad \frac{p \rightsquigarrow q \quad q \rightsquigarrow r}{p \rightsquigarrow r}$$

**Fig. 1.** Inference rules for computing $Q^e_\infty$.

*Example 4.* We obtain $3 \rightsquigarrow 5$ and $4 \rightsquigarrow 5$ by applying the first inference rule to the transition rule $\bot\mathsf{f}(3,4) \to 5$. Similarly, $\bot\mathsf{g}(5) \to 5$ gives rise to $5 \rightsquigarrow 5$. Since $\mathcal{A}$ has no epsilon transitions, no further inferences can be made. It follows that $Q^e_\infty = \{5\}$.

We call a term in $\mathcal{T}(\{\bot\} \times \mathcal{F})$ *right-only.* A term in $\mathcal{T}((\{\bot\} \times \mathcal{F}) \cup \{\square\})$ with exactly one occurrence of the hole $\square$ is a right-only context.

**Definition 4.** *We denote the composition of $\to_{\Delta_{\neg\epsilon}}$ and $\to^*_{\Delta_\epsilon}$ by $\twoheadrightarrow_\Delta$.*

The proof of the next lemma is straightforward. Note that the relations $\to^*_\Delta$ and $\twoheadrightarrow^*_\Delta$ do not coincide on *mixed* terms, involving function symbols and states.

**Lemma 5.** *Let $C$ be a ground context. We have $C[p] \to^*_\Delta q$ if and only if $p \to^*_\Delta p'$ and $C[p'] \twoheadrightarrow^*_\Delta q$ for some state $p'$.* ☑

**Lemma 6.** $Q_\infty \subseteq Q^e_\infty$

*Proof.* We start by proving the following claim:

$$\text{if } C[p] \twoheadrightarrow^*_\Delta q \text{ and } C \text{ is a non-empty right-only context then } p \rightsquigarrow q \qquad (*)$$

We use induction on the structure of $C$. If $C = \square$ there is nothing to show. Suppose $C = \bot f(t_1,\ldots,C',\ldots,t_n)$ where $C'$ is the $i$-th subterm of $C$. The sequence $C[p] \twoheadrightarrow^*_\Delta q$ can be rearranged as $C[p] = \bot f(t_1,\ldots,C'[p],\ldots,t_n) \twoheadrightarrow^*_\Delta \bot f(q_1,\ldots,q_n) \to_\Delta q' \to^*_\Delta q$. We obtain $q_i \rightsquigarrow q'$ and subsequently $q_i \rightsquigarrow q$ by using the inference rules in Figure 1. If $C' = \square$ then $p = q_i$ and if $C' \neq \square$ then the induction hypothesis yields $p \rightsquigarrow q_i$ and thus $p \rightsquigarrow q$ by transitivity. This concludes the proof of $(*)$. ☑

Assume $q \in Q_\infty$, so there exist infinitely many terms $t$ such that $\langle\bot,t\rangle \to^*_\Delta q$. Since the signature is finite, there exist terms of arbitrary height. Thus there exists an arbitrary but fixed term $t$ such that the height of $t$ is greater than the number of states of $Q$. Write $t = f(t_1,\ldots,t_n)$. Since the height of $t$ is greater than the number of the states in $Q$, there exist a subterm $s$ of $t$, a state $p$, and contexts $C_1$ and $C_2 \neq \square$ such that

1. $\langle\bot,t\rangle = C_1[C_2[\langle\bot,s\rangle]]$,
2. $\langle\bot,s\rangle \to^*_\Delta p$,
3. $C_2[p] \to^*_\Delta p$, and

4. $C_1[p] \to_\Delta^* q$.

From Lemma 5 we obtain a state $q'$ such that $p \to_\Delta^* q'$ and $C_2[q'] \twoheadrightarrow_\Delta^* p$. Hence $q' \rightsquigarrow p$ by ($*$). We obtain $q' \rightsquigarrow q'$ from $q' \rightsquigarrow p$ in connection with the inference rule for epsilon transitions. We perform a case analysis of the context $C_1$.

– If $C_1 = \Box$ then $p \to_\Delta^* q$ and thus $q' \rightsquigarrow q$ follows from $q' \rightsquigarrow p$ in connection with the inference rule for epsilon transitions. Hence $q \in Q_\infty^e$.
– If $C_1 \neq \Box$ then Lemma 5 yields a state $q''$ such that $p' \to_\Delta^* q''$ and $C_1[q''] \twoheadrightarrow_\Delta^* q$. Hence $q'' \rightsquigarrow q$ by ($*$). We also have $C_2[q'] \twoheadrightarrow_\Delta^* q''$ and thus $q' \rightsquigarrow q''$ by ($*$). We obtain $q' \rightsquigarrow q$ from the transitivity rule. Hence also in this case we obtain $q \in Q_\infty^e$. ☑

For the following lemma, we need the fact that $\mathcal{A}$ can be assumed to be trim, so every state is productive and reachable. We may do so because Theorem 1 talks about regular relations, and any automaton that accepts the same language as $\mathcal{A}$ will witness the fact that the given relation $\circ$ is regular.

**Lemma 7.** *$Q_\infty^e \subseteq Q_\infty$, provided that $\mathcal{A}$ is trim.*

*Proof.* In connection with the fact that $\mathcal{A}$ accepts $\circ \subseteq \mathcal{T}(\mathcal{F}) \times \mathcal{T}(\mathcal{F})$, trimness of $\mathcal{A}$ entails that any run $t \to_\Delta^* q$ is embedded into an accepting run $C[t] \to_\Delta^* C[q] \to_\Delta^* q_f \in Q_f$. So $C[t] = \langle u, v\rangle$ for some $(u, v) \in \circ$, and hence $t$ must be a well-formed term. Moreover, if $\mathsf{root}(t) = \bot f$ for some $f \in \mathcal{F}$ then $t = \langle \bot, u\rangle$ for some term $u \in \mathcal{T}(\mathcal{F})$. We now show the converse of claim ($*$) in the proof of Lemma 6 for the relation $\to_\Delta^*$:

$$\text{if } p \rightsquigarrow q \text{ then } C[p] \to_\Delta^* q \text{ for some ground right-only context } C \neq \Box \qquad (**)$$

We prove the claim by induction on the derivation of $p \rightsquigarrow q$. First suppose $p \rightsquigarrow q$ is derived from the transition rule $\bot f(p_1, \dots, p_i, \dots, p_n) \to q$ in $\Delta$ with $p_i = p$. Because all states are reachable by well-formed terms, there exist terms $t_1, \dots, t_n \in \mathcal{T}(\mathcal{F})$ such that $\langle \bot, t\rangle \to_\Delta^* p_i$ for all $1 \leqslant i \leqslant n$. Let $C_1 = \bot f(\langle \bot, t_1\rangle, \dots, \Box, \dots, \langle \bot, t_n\rangle)$ where the hole is the $i$-th argument. We have $C_1[p] \to_\Delta^* \bot f(p_1, \dots, p_i, \dots, p_n) \to_\Delta q$. Next suppose $p \rightsquigarrow q$ is derived from $p \rightsquigarrow q'$ and $q' \to_\Delta q$. The induction hypothesis yields a ground right-only context $C \neq \Box$ such that $C[p] \to_\Delta^* q'$. Hence also $C[p] \to_\Delta^* q$. Finally, suppose $p \rightsquigarrow q$ is derived from $p \rightsquigarrow r$ and $r \rightsquigarrow q$. The induction hypothesis yields non-empty ground right-only contexts $C_1$ and $C_2$ such that $C_1[p] \to_\Delta^* r$ and $C_2[r] \to_\Delta^* q$. Hence $C[p] \to_\Delta^* q$ for the context $C = C_2[C_1]$. This concludes the proof of ($**$). ☑

Now let $q \in Q_\infty^e$. So there exists a state $p$ such that $p \rightsquigarrow p$ and $p \rightsquigarrow q$. Using ($**$), we obtain non-empty ground right-only contexts $C_1$ and $C_2$ such that $C_1[p] \to_\Delta^* p$ and $C_2[p] \to_\Delta^* q$. Since all states are reachable, there exists a ground term $t \in \mathcal{T}(\mathcal{F}^{(2)})$ such that $t \to_\Delta^* p$. Hence $C_2[t] \to_\Delta^* q$ and, by the observation made at the beginning of the proof, $C_2[t]$ is a well-formed term. Since $C_2$ is right-only, it follows that $t = \langle \bot, u\rangle$ for some term $u \in \mathcal{T}(\mathcal{F})$. Now consider the infinitely many terms $t_n = C_2[C_1^n[t]]$ for $n \geqslant 0$. We have $t_n \to_\Delta^* q$ and $t_n$ is right-only by construction. Hence $q \in Q_\infty$. ☑

**Corollary 1.** *$Q^e_\infty = Q_\infty$, provided that $\mathcal{A}$ is trim.* □

## 5 Normal Form Predicate

The normal form predicate NF can be defined in the first-order theory of rewriting as

$$\mathsf{NF}(t) \iff \neg\,\exists\, u\,(t \to u)$$

and this gives rise to the following procedure:

1. An $\mathrm{RR}_2$ automaton is constructed that accepts the encoding of the rewrite relation $\to$.
2. The $\mathrm{RR}_2$ automaton of step 1 is *projected* into a tree automaton that accepts the set of reducible ground terms.
3. Complementation is applied to the automaton of step 2 to obtain a tree automaton that accepts the set of ground normal forms.

Since projection may transform a deterministic tree automaton into a non-deterministic one, this is inefficient. In this section we provide a direct construction of a tree automaton that accepts the set of ground normal forms of a left-linear TRS, which goes back to Comon [5], and present a formalized correctness proof. Throughout this section $\mathcal{R}$ is assumed to be left-linear.

We start with defining some preliminary concepts.

**Definition 5.** *Given a signature $\mathcal{F}$, we write $\mathcal{F}_\bot$ for the extension of $\mathcal{F}$ with a fresh constant symbol $\bot$. Given $t \in \mathcal{T}(\mathcal{F},\mathcal{V})$, $t^\bot$ denotes the result of replacing all variables in $t$ by $\bot$:*

$$x^\bot = \bot \qquad\qquad f(t_1,\ldots,t_n)^\bot = f(t_1^\bot,\ldots,t_n^\bot)$$

*We define the partial order $\leqslant$ on $\mathcal{T}(\mathcal{F}_\bot)$ as the least congruence that satisfies $\bot \leqslant t$ for all terms $t \in \mathcal{T}(\mathcal{F}_\bot)$:*

$$\frac{}{\bot \leqslant t} \qquad\qquad \frac{t_1 \leqslant u_1 \quad \cdots \quad t_n \leqslant u_n}{f(t_1,\ldots,t_n) \leqslant f(u_1,\ldots,u_n)}$$

*The partial map $\uparrow\colon \mathcal{T}(\mathcal{F}_\bot) \times \mathcal{T}(\mathcal{F}_\bot) \to \mathcal{T}(\mathcal{F}_\bot)$ is defined as follows:*

$$\bot \uparrow t = t \uparrow \bot = t \qquad f(t_1,\ldots,t_n) \uparrow f(u_1,\ldots,u_n) = f(t_1 \uparrow u_1,\ldots,t_n \uparrow u_n)$$

It is not difficult to show that $t \uparrow u$ is the least upper bound of comparable terms $t$ and $u$.

**Definition 6.** *Let $\mathcal{R}$ be a TRS over a signature $\mathcal{F}$. We write $T^\bot$ for the set $\{t^\bot \mid t \lhd \ell$ for some $\ell \to r \in \mathcal{R}\} \cup \{\bot\}$. The set $T_\uparrow$ is obtained by closing $T^\bot$ under $\uparrow$.*

*Example 5.* Consider the TRS $\mathcal{R}$ consisting of following rules:

$$\mathsf{h}(\mathsf{f}(\mathsf{g}(\mathsf{a}), x, y)) \to \mathsf{g}(\mathsf{a}) \qquad \mathsf{g}(\mathsf{f}(x, \mathsf{h}(x), y))) \to x \qquad \mathsf{h}(\mathsf{f}(x, y, \mathsf{h}(\mathsf{a}))) \to \mathsf{h}(x)$$

We start by collecting the subterms of the left-hand sides:

$$T^{\perp} = \{\perp, \mathsf{a}, \mathsf{g}(\mathsf{a}), \mathsf{h}(\perp), \mathsf{h}(\mathsf{a}), \mathsf{f}(\mathsf{g}(\mathsf{a}), \perp, \perp), \mathsf{f}(\perp, \mathsf{h}(\perp), \perp), \mathsf{f}(\perp, \perp, \mathsf{h}(\mathsf{a}))\}$$

Closing $T^{\perp}$ under $\uparrow$ adds the following terms:

$$\begin{aligned} \mathsf{f}(\mathsf{g}(\mathsf{a}), \perp, \perp) \uparrow \mathsf{f}(\perp, \mathsf{h}(\perp), \perp) &= \mathsf{f}(\mathsf{g}(\mathsf{a}), \mathsf{h}(\perp), \perp) \\ \mathsf{f}(\perp, \perp, \mathsf{h}(\mathsf{a})) \uparrow \mathsf{f}(\perp, \mathsf{h}(\perp), \perp) &= \mathsf{f}(\perp, \mathsf{h}(\perp), \mathsf{h}(\mathsf{a})) \\ \mathsf{f}(\mathsf{g}(a), \mathsf{h}(\perp), \perp) \uparrow \mathsf{f}(\perp, \mathsf{h}(\perp), \mathsf{h}(\mathsf{a})) &= \mathsf{f}(\mathsf{g}(\mathsf{a}), \mathsf{h}(\perp), \mathsf{h}(\mathsf{a})) \end{aligned}$$

**Lemma 8.** *The set $T_{\uparrow}$ is finite.*

*Proof.* If $t \uparrow u$ is defined then $\mathcal{P}\mathsf{os}(t \uparrow u) = \mathcal{P}\mathsf{os}(t) \cup \mathcal{P}\mathsf{os}(u)$. It follows that the positions of terms in $T_{\uparrow} \setminus T^{\perp}$ are positions of terms in $T^{\perp}$. Since $T^{\perp}$ is finite, there are only finitely many such positions. Hence the finiteness of $T_{\uparrow}$ follows from the finiteness of $\mathcal{F}$. □

Although the above proof is simple enough, we formalized the proof below which is based on a concrete algorithm to compute $T_{\uparrow}$. Actually, the algorithm presented below is based on a general saturation procedure, which is of independent interest.

**Definition 7.** *Let $f \colon U \times U \to U$ be a (possibly partial) function and let $S$ be a finite subset of $U$. The* closure *$C_f(S)$ is the least extension of $S$ with the property that $f(a,b) \in C_f(S)$ whenever $a, b \in C_f(S)$ and $f(a,b)$ is defined.*

The following lemma provides a sufficient condition for closures to exist. The proof gives a concrete algorithm to compute the closure.

**Lemma 9.** *If $f$ is a total, associative, commutative, and idempotent function then $C_f(S)$ exists and is finite.*

*Proof.* A straightforward induction proof reveals that for every $a \in C_f(S)$ there exist elements $a_1, \ldots, a_n \in S$ such that $a = f(a_1, f(a_2, \ldots f(a_{n-1}, a_n) \ldots))$. Select an arbitrary element $b \in S$. If $b$ is among $a_1, \ldots, a_n$ then, using the properties of $f$, we obtain $a \in \{f(b,c) \mid c \in C_f(S \setminus \{b\})\}$. If $b$ is not among $a_1, \ldots, a_n$ then $a \in C_f(S \setminus \{b\})$. Hence

$$C_f(S) = C_f(S \setminus \{b\}) \cup \{b\} \cup \{f(b,c) \mid c \in C_f(S \setminus \{b\})\}$$

for every $b \in S$. Since $S$ is finite, this gives rise to an iterative algorithm to compute $C_f(S)$, which is given in Listing 5. In each iteration only finitely many elements are added. Hence $C_f(S)$ is finite. ☑

```
saturate(S):
    I ← ∅
    for all x ∈ S do
        I ← {x} ∪ I ∪ {f(x, y) | y ∈ I}
    return I
```

**Listing 1.** Iterative closure algorithm.

Since our function $\uparrow$ is partial, we need to lift it to a total function that preserves associativity and commutativity. In our abstract setting this entails finding a binary predicate $P$ on $U$ such that $f(a,b)$ is defined if $P(a,b)$ holds. In addition, the following properties need to be fulfilled:

– $P$ is reflexive and symmetric,
– if $P(a, f(b,c))$ and $P(b,c)$ hold then $P(a,b)$ and $P(f(a,b),c)$ hold as well, for all $a,b,c \in U$.

For the details we refer to the formalization.

**Definition 8.** *The tree automaton $\mathcal{A}_{\mathsf{NF}(\mathcal{R})} = (\mathcal{F}, Q, Q_f, \Delta)$ is defined as follows: $Q = Q_f = T_\uparrow$ and $\Delta$ consists of all transition rules*

$$f(p_1, \dots, p_n) \to q$$

*such that $f(p_1,\dots,p_n)$ is no redex and $q$ is the maximal element of $Q$ satisfying $q \leqslant f(p_1,\dots,p_n)$.*[4]

*Example 6.* For the TRS $\mathcal{R}$ of Example 5, the tree automaton $\mathcal{A}_{\mathsf{NF}(\mathcal{R})}$ consists of the following transition rules:

$$\mathsf{a} \to 1 \qquad \mathsf{g}(p) \to \begin{cases} 2 & \text{if } p = 1 \\ 0 & \text{if } p \notin \{1,6,9,10\} \end{cases} \qquad \mathsf{h}(p) \to \begin{cases} 4 & \text{if } p = 1 \\ 3 & \text{if } p \notin \{1,8,10\} \end{cases}$$

$$\mathsf{f}(p,q,r) \to \begin{cases} 5 & \text{if } p = 2,\ q \notin \{3,4\} \\ 6 & \text{if } p \neq 2,\ q \in \{3,4\},\ r \neq 4 \\ 7 & \text{if } q \notin \{3,4\},\ r = 4 \\ 8 & \text{if } p = 2,\ q \in \{3,4\},\ r \neq 4 \\ 9 & \text{if } p \neq 2,\ q \in \{3,4\},\ r = 4 \\ 10 & \text{if } p = 2,\ q \in \{3,4\},\ r = 4 \\ 0 & \text{otherwise} \end{cases}$$

Here we use the following abbrevations:

| | | | |
|---|---|---|---|
| $0 = \bot$ | $3 = \mathsf{h}(\bot)$ | $6 = \mathsf{f}(\bot, \mathsf{h}(\bot), \bot)$ | $8 = \mathsf{f}(\mathsf{g}(\mathsf{a}), \mathsf{h}(\bot), \bot)$ |
| $1 = \mathsf{a}$ | $4 = \mathsf{h}(\mathsf{a})$ | $7 = \mathsf{f}(\bot, \bot, \mathsf{h}(\mathsf{a}))$ | $9 = \mathsf{f}(\bot, \mathsf{h}(\bot), \mathsf{h}(\mathsf{a}))$ |
| $2 = \mathsf{g}(\mathsf{a})$ | $5 = \mathsf{f}(\mathsf{g}(\mathsf{a}), \bot, \bot)$ | | $10 = \mathsf{f}(\mathsf{g}(\mathsf{a}), \mathsf{h}(\bot), \mathsf{h}(\mathsf{a}))$ |

[4] Since states are terms from $T_\infty$ here, Definition 5 applies.

As can be seen from the above example, the tree automaton $\mathcal{A}_{\mathsf{NF}(\mathcal{R})}$ is not completely defined. Unlike the construction in [5], we do not have an additional state that is reached by all reducible ground terms.

Before proving that $\mathcal{A}_{\mathsf{NF}(\mathcal{R})}$ accepts the ground normal forms of $\mathcal{R}$, we first show that $\mathcal{A}_{\mathsf{NF}(\mathcal{R})}$ is well-defined, which amounts to showing that for every $f(p_1, \ldots, p_n)$ with $f \in \mathcal{F}$ and $p_1, \ldots, p_n \in T_\uparrow$ the set of states $q$ such that $q \leqslant f(p_1, \ldots, p_n)$ has a maximum element with respect to the partial order $\leqslant$.

**Lemma 10.** *For every term $t \in T_\uparrow$ the set $\{s \in T_\uparrow \mid s \leqslant t\}$ has a unique maximal element.*

*Proof.* Let $S = \{s \in T_\uparrow \mid s \leqslant t\}$. Because $\bot \leqslant t$ and $\bot \in T_\uparrow$, $S \neq \varnothing$. If $s_1, s_2 \in T$ then $s_1 \leqslant t$ and $s_2 \leqslant t$ and thus $s_1 \uparrow s_2$ is defined and satisfies $s_1 \uparrow s_2 \leqslant t$. Since $T_\uparrow$ is closed under $\uparrow$, $s_1 \uparrow s_2 \in T_\uparrow$ and thus $s_1 \uparrow s_2 \in P$. Consequently, $S$ has a unique maximal element. □

The next lemma is a trivial consequence of the fact that $\mathcal{A}_{\mathsf{NF}(\mathcal{R})}$ has no epsilon transitions.

**Lemma 11.** *The tree automaton $\mathcal{A}_{\mathsf{NF}(\mathcal{R})}$ is deterministic.*

**Lemma 12.** *If $t \in \mathcal{T}(\mathcal{F})$ with $t \to_\Delta^* q$ and $s^\bot \leqslant t^\bot$ for a proper subterm $s$ of some left-hand side of $\mathcal{R}$ then $s^\bot \leqslant q$.*

*Proof.* We use structural induction on $t$. Let $t = f(t_1, \ldots, t_n)$. We have $t \to_\Delta^* f(q_1, \ldots, q_n) \to_\Delta q$. We procede by case analysis on $s$. If $s$ is a variable then $s^\bot = \bot$ and, as $\bot$ is minimal in $\leqslant$, we obtain $s^\bot \leqslant q$. Otherwise we must have $\mathsf{root}(s) = f$ from the assumption $s^\bot \leqslant t^\bot$. So we may write $s = f(s_1, \ldots, s_n)$. The induction hypothesis yields $s_i^\bot \leqslant q_i$ for all $1 \leqslant i \leqslant n$. Hence $s^\bot = f(s_1^\bot, \ldots, s_n^\bot) \leqslant f(q_1, \ldots, q_n)$. Additionally we have $s^\bot \in Q$ by Definition 8 as $s$ is a proper subterm of a left-hand side of $\mathcal{R}$. Since $f(q_1, \ldots, q_n) \to q$ is a transition rule, we obtain $f(s_1, \ldots, s_n)^\bot \leqslant q$ from the maximality of $q$.

Using the previous result we can prove that no redex of $\mathcal{R}$ reaches a state in $\mathcal{A}_{\mathsf{NF}(\mathcal{R})}$.

**Lemma 13.** *If $t \in \mathcal{T}(\mathcal{F})$ is a redex then $t \to_\Delta^* q$ for no state $q \in T_\uparrow$.*

*Proof.* We have $\ell^\bot \leqslant t$ for some left-hand side $\ell$ of $\mathcal{R}$. For a proof by contradiction, assume $t \to_\Delta^* q$. Write $t = f(t_1, \ldots, t_n)$. We have $t \to_\Delta^* f(q_1, \ldots, q_n) \to_\Delta q$ and obtain $\ell^\bot \leqslant f(q_1, \ldots, q_n)$ by a case analysis on $\ell$ and Lemma 12. Therefore the transition rule $f(q_1, \ldots, q_n) \to_\Delta q$ cannot exist by Definition 8.

**Lemma 14.** *If $t \to_\Delta^* q$ and $t \in \mathcal{T}(\mathcal{F})$ then $q \leqslant t$.*

*Proof.* We use structural induction on $t$. Let $t = f(t_1, \ldots, t_n)$. We have $t \to_\Delta^* f(q_1, \ldots, q_n) \to_\Delta^* q$. The induction hypothesis yields $q_i \leqslant t_i$ for all $1 \leqslant i \leqslant n$ and thus also $f(q_1, \ldots, q_n) \leqslant f(t_1, \ldots, t_n)$. We have $q \leqslant f(q_1, \ldots, q_n)$ by Definition 8 and thus $q \leqslant t$ by the transitivity of $\leqslant$.

**Lemma 15.** *If* $t \in \mathsf{NF}(\mathcal{R})$ *then* $t \to_{\Delta}^{*} q$ *for some state* $q \in T_{\uparrow}$.

*Proof.* We use structural induction on $t$. Let $t = f(t_1, \dots, t_n)$. Since $t_1, \dots, t_n \in \mathsf{NF}(\mathcal{R})$ we obtain $f(t_1, \dots, t_n) \to_{\Delta}^{*} f(q_1, \dots, q_n)$ from the induction hypothesis. Suppose $f(q_1, \dots, q_n)$ is a redex, so $l^{\perp} \leqslant f(q_1, \dots, q_n)$ for some left-hand side $\ell$ of $\mathcal{R}$. From Lemma 14 we obtain $q_i \leqslant t_i$ for all $1 \leqslant i \leqslant n$ and thus $f(q_1, \dots, q_n) \leqslant f(t_1, \dots, t_n)$. Hence $\ell^{\perp} \leqslant f(t_1, \dots, t_n)$. This however contradicts the assumption that $t$ is a normal form. (Here we need left-linearity of $\mathcal{R}$.) Therefore $f(q_1, \dots, q_n)$ is no redex and thus, using Lemma 10, there exists a transition $f(q_1, \dots, q_n) \to q$ in $\Delta$ and thus $t \to_{\Delta}^{*} q$. ☑

**Theorem 4.** *If* $\mathcal{R}$ *is a left-linear TRS then* $L(\mathcal{A}_{\mathsf{NF}(\mathcal{R})}) = \mathsf{NF}(\mathcal{R})$.

*Proof.* Let $t \in \mathcal{T}(\mathcal{F})$. If $t \in \mathsf{NF}(\mathcal{R})$ then $t \to_{\Delta}^{*} q$ for some state $q \in T_{\uparrow}$ by Lemma 15. Since all states in $T_{\uparrow}$ are final, $t \in L(\mathcal{A}_{\mathsf{NF}(\mathcal{R})})$. ☑

Next assume $t \notin \mathsf{NF}(\mathcal{R})$. Hence $t = C[s]$ for some redex $s$. According to Lemma 13 $s$ does not reach a state in $\mathcal{A}_{\mathsf{NF}(\mathcal{R})}$. Hence also $t$ cannot reach a state and thus $t \notin L(\mathcal{A}_{\mathsf{NF}(\mathcal{R})})$. ☑

## 6 Conclusion and Future Work

In this paper we presented formalized correctness proofs of the regularity of the infinity and normal form predicates in the first-order theory of rewriting. For the former we also provided an executable version, which is important for checking certificates that will be provided in a future version of FORT. Our results are an important step towards the ultimate goal of proving the correctness of the decisions reported by FORT, but much work remains to be done. We are developing a certification language which reflects the high-level proof steps in the decision procedure for the full first-order theory of rewriting. This language will be independent of FORT. In particular, details of the intermediate tree automata computed by FORT will not be part of certificates. This keeps the certificates small and avoids having to implement a verified (and expensive) equivalence check on tree automata. We will provide executable Isabelle code for each of the constructs in the certification language, and so this involves replaying the automata constructions in Isabelle.

We conclude the paper by providing some details of the size of our formalization in Table 1.

*Acknowledgments.* We thank Bertram Felgenhauer and T. V. H. Prathamash for contributions in the early stages of this work. The comments by the reviewers helped to improve the presentation of the paper.

**Table 1.** Formalization data.

| theory | lines | facts | defs |
|---|---|---|---|
| Saturation.thy | 233 | 22 | 3 |
| Tree_Automata_Pumping.thy | 371 | 40 | 2 |
| NF.thy | 404 | 40 | 7 |
| RR2_Infinite.thy | 603 | 48 | 5 |
| RR2_Infinite_Impl.thy | 240 | 14 | 2 |
| total | 1851 | 164 | 19 |

**Open Access** This chapter is licensed under the terms of the Creative Commons Attribution 4.0 International License (http://creativecommons.org/licenses/by/4.0/), which permits use, sharing, adaptation, distribution and reproduction in any medium or format, as long as you give appropriate credit to the original author(s) and the source, provide a link to the Creative Commons license and indicate if changes were made.

The images or other third party material in this chapter are included in the chapter's Creative Commons license, unless indicated otherwise in a credit line to the material. If material is not included in the chapter's Creative Commons license and your intended use is not permitted by statutory regulation or exceeds the permitted use, you will need to obtain permission directly from the copyright holder.
